# Familial Risks between Pernicious Anemia and Other Autoimmune Diseases in the Population of Sweden

**DOI:** 10.1155/2021/8815297

**Published:** 2021-01-12

**Authors:** Xinjun Li, Hauke Thomsen, Kristina Sundquist, Jan Sundquist, Asta Försti, Kari Hemminki

**Affiliations:** ^1^Center for Primary Health Care Research, Lund University, Malmö, Sweden; ^2^Division of Molecular Genetic Epidemiology, German Cancer Research Center (DKFZ), 69120 Heidelberg, Germany; ^3^Genewerk GmbH, Heidelberg, Germany; ^4^Department of Family Medicine and Community Health, Department of Population Health Science and Policy, Icahn School of Medicine at Mount Sinai, New York, USA; ^5^Center for Community-based Healthcare Research and Education (CoHRE), Department of Functional Pathology, School of Medicine, Shimane University, Matsue, Japan; ^6^Hopp Children's Cancer Center (KiTZ), Heidelberg, Germany; ^7^Division of Pediatric Neurooncology, German Cancer Research Center (DKFZ), German Cancer Consortium (DKTK), Heidelberg, Germany; ^8^Division of Cancer Epidemiology, German Cancer Research Centre (DKFZ), 69120 Heidelberg, Germany; ^9^Faculty of Medicine and Biomedical Center in Pilsen, Charles University in Prague, 30605 Pilsen, Czech Republic

## Abstract

**Background:**

Pernicious anemia (PA) is an autoimmune disease (AID) which is caused by lack of vitamin B12 (cobalamin) due to its impaired uptake. PA is a multifactorial disease which is associated with a number of other AID comorbidities and which is manifested as part of autoimmune polyglandular syndrome. Due to the shortage of family studies on PA, we planned to address the problem by assessing familial risks for concordant PA between family members and for discordant PA in families of other AID patients.

**Methods:**

We collected data on patients diagnosed with AIDs from the Swedish hospitals and family data from a population register. We calculated standardized incidence ratios (SIRs) in families for concordant and discordant risks.

**Results:**

The number of PA patients in the offspring generation (for which the familial risk was calculated) was 7701; 278 (3.6%) patients had a family history of PA. The population prevalence of PA was 0.9/1000. The familial risk for PA was 3.88 when any first-degree relative was the proband, equal for men and women. The familial risk was two times higher between siblings than between offspring and parents which may be due to complex genetic background. Associations of PA with 14 discordant AIDs were significant; these included some AIDs that have previously been described as comorbidities in PA patients and several yet unreported associations, including rheumatoid arthritis and other AIDs.

**Conclusions:**

The familial risks for PA were high suggesting multifactorial genetic etiology. The results call for further population-level studies to unravel mechanisms of familial PA which may help to understand the etiology of this disease.

## 1. Introduction

Pernicious anemia (PA) is an autoimmune disease (AID) which is diagnosed because of anemia caused by lack of vitamin B12 (cobalamin) in the blood [1]. Vitamin B12 deficiency results from its impaired uptake due to the lack of intrinsic factor (IF). IF is a glycoprotein binding vitamin B12, which is produced by the parietal cells of the gastric mucosa and enables absorption of the vitamin B12-intrinsic factor complex by cublilin receptors at the terminal ileum [1, 2]. PA is a multifactorial disease which arises because of the interplay between nature and nurture; it manifests as the commonest cause of vitamin B12 deficiency in the world and is frequently associated with chronic atrophic gastritis [3]. The prevalence of PA in the USA is about one per 1000 people, but in those over the age of 60 years, it is about 2% [2, 4]. Women are more commonly affected than men. With proper treatment, most people live normal lives [1].

Autoantibodies in PA attack both IF and the gastric parietal cells producing IF. They may block either the vitamin B12 binding site or the ileal mucosal receptor [2]. The autoimmune cascade in parietal cells begins with the activation of gastric dendritic cells, which in turn activate CD4+ T cell lymphocytes, targeting the parietal cell proton pump ATPase [2]. PA is associated with a number of other AID comorbidities and is thus a true polyautoimmune disease [5]. PA is strongly associated with a personal history of autoimmune thyroid diseases, and it is a part of autoimmune polyglandular syndrome (APS) [6–8]. PA is also encountered with other AID comorbidities, such as type 1 diabetes, rheumatoid arthritis, vitiligo, and Addison disease [1, 9–12]. Additionally, patients with PA are at increased risk of gastric cancer and hematological malignancies and possibly also esophageal cancer [13–18].

Although many comorbidities are well characterized in PA patients, disease risks between family members are less known [19]. Due to the sparsity of population-based family studies in PA, we used Swedish hospital and population register data to characterize familial clustering of concordant (PA) and discordant (PA and other AIDs) disease. Concordant risks were assessed separately for offspring of affected parents and for siblings in order to draw conclusions about the mode of association. The discordant analysis was conducted bidirectionally: first, the relative risk was calculated for any other AID in families of PA, followed by the relative risk for PA in families with other AIDs. Additionally, incidence and prevalence rates for hospitalized PA patients are reported.

## 2. Materials and Methods

AID patients (*N* = 896,270) were identified from the Swedish Hospital Discharge Register (years 1964 through 2015, full national coverage from 1986 onwards) and the Outpatient Register (2001 through 2015). Only the first recorded AID was considered in the analyses. Various revisions of the International Classification of Diseases (ICD) codes were used for AIDs as described elsewhere [6]. The list of all 43 AIDs in this study is given in Supplementary Table 1, with patient numbers and percent shares for each among a total of 896,270 patients. Family relationships were obtained from the Multigeneration Register, containing the Swedish population in families and spanning more than a century [20]. As family members, only first-degree relatives of offspring-parent pairs and siblings in “the offspring generation” were considered; “the offspring generation” was born after 1931 and “the parental generation” included parents of the offspring. By year 2015, the offspring generation reached age 83 years; siblings can be defined only in the offspring generation. For the parental generation, there was no age limit. Information from the registers was linked at the individual level via the national 10-digit civic registration number. In the linked dataset, civic registration numbers were replaced with serial pseudonymized numbers to ensure the integrity of all individuals.

Standardized incidence ratios (SIRs) were calculated for the offspring generation (8.9 million with 7701 PA patients, Table 1) as the ratio of observed to expected number of cases. The observed number was the number of PA patients in the offspring generation, whose family members were diagnosed with PA (for concordant association) or with another AID (for discordant association). The expected numbers were calculated for all individuals in the Swedish offspring generation without a first-degree family history of a specific AID, and the rates were standardized by 5-year age, gender, period (5-year groups), socioeconomic status, and residential area. The 95% confidence interval (95% CI) of the SIR was calculated assuming a Poisson distribution. Separate SIRs were calculated for offspring when any first-degree relative or defined family members (only parent, only sibling, or parent and sibling) were probands, i.e., they were diagnosed with concordant AID. Case numbers were somewhat lower in the first type of analysis (first-degree family members) because when any family member was first diagnosed with an AID other than PA, the family was excluded. In analysis of discordant AIDs, bidirectional (e.g., PA-Hashimoto and Hashimoto-PA) associations were considered. Using the common epidemiological practice, differences between two SIRs were considered significant when their 95% CIs did not overlap, as shown in the tables.

Age-specific incidence and age-specific prevalence rates in the Swedish population were calculated for the period 1964–2015 using the standard European population as a reference.

The study was approved by the Regional Ethical Review Board in Lund February 6, 2013 (Reference 2012/795). All guidelines of the Helsinki Declaration were followed. The study was conducted in accordance with the approved guidelines with explicit statement that no informed consent was required. The study is national register-based study on anonymous personal data.

## 3. Results

The number of PA patients in the offspring generation (to which risks were calculated) was 7701 with a mean diagnostic age (i.e., first hospital contact) of 47.7 years; considering also the parents, the total number was 35,906 of whom 42.3% were men (Table 1). Offspring with a family history (parents or siblings with PA) numbered 278 (3.6%). The total AID population amounted to 896,270 patients; thus, PA accounted for 4.0% of hospitalized AIDs (Supplementary Table 1).

The age-specific incidence for PA is shown in Supplementary Figure 1, describing a higher incidence for women than for men in the middle ages, and, for both sexes, a vastly increasing rate towards the maximum at age 80–84 years, reaching 56/100,000 for both sexes. The overall population incidence was 8.3/100,000, and it was 7.5/100,000 for men and 9.1/100, 000 for women. The corresponding prevalence rates are shown in Supplementary Figure 2; the overall population prevalence was 0.9/1000, lower for men (0.8/1000) than for women (1.1/1000).

### 3.1. Concordant Familial Risks

Familial risks for PA are shown in Table 2 for offspring whose first-degree relatives (parents or siblings) were diagnosed with PA. The familial risk for PA was 3.88 when any first-degree relatives were probands, and it was marginally higher for men (4.24) than women (3.58, with overlapping 95% CIs). SIRs were higher when siblings (6.43) than when parents were probands (3.08, nonoverlapping 95% CIs). Note that the sum of affected offspring was 250 in the top row and 278 in the bottom row; the reason was that for the first-degree relatives, more families were removed because a non-PA AID was first diagnosed in a family member (see Methods). Spousal risk for PA was 1.21 (*N* = 100, 95% CI 0.99–1.47).

### 3.2. Discordant Familial Risks

We analyzed familial risks between PA and all 42 other AIDs, and the significant discordant associations are shown in Table 3. Associations with 14 AIDs were significant, and of these, significant bidirectional associations were noted for 10 AID pairs (considering both genders). The highest risks for PA were with Addison disease (2.45) and Crohn disease (1.70); in the reverse analysis, Addison disease (1.97) and type I diabetes (1.74) associated most strongly with PA. Comparing the two bidirectional SIRs for both genders, the risk of PA with Crohn disease (1.70) was significantly higher than the risk for Crohn disease with PA (1.24). The association of polymyalgia rheumatic risk with PA (1.27) was also higher than the reverse risk (0.82). Associations with Hashimoto thyroiditis and Sjögren syndrome were significant only for women, but no sex difference was significant (i.e., 95% CIs overlapped). Also, discordant spousal risks were analyzed, but none were significant (data not shown). Data for the remaining 28 AIDs, lacking significant associations with PA, are shown in Supplementary Table 2.

## 4. Discussion

The present study covered 35,906 patients diagnosed with PA in Swedish hospitals, which accounted for 4.0% of all diagnosed AIDs in the country. Epidemiological data on PA are scanty, but our prevalence estimate of 0.9/1000 is close to the rate reported for USA and some other countries [4, 19]. Our age-specific prevalence and incidence data confirm the strong age dependence of PA.

To our knowledge, this is the first family study on concordant PA and on all main types of other AIDs. The results showed that the concordant familial clustering was observed for 3.6% of PA patients. The familial risk for first-degree relatives was 3.88, and the SIR was only marginally higher for men than women. There was a significant difference in familial risk when siblings (6.43) compared to parents (3.08) were probands, which is usually considered to suggest either recessive inheritance or influence by shared childhood environment. The genetic basis of PA is not fully characterized, but several genes have been identified which influence vitamin B12 serum levels, including FUT2, FUT6, CUBN, TCN1, MUT, and CLYBL [21]. Human leucocyte antigen (HLA) haplotypes may influence the tissues in which autoimmune processes develop, and associations of PA with several HLA DR haplotypes have been reported [19, 22]. PA is part of autoimmune polyglandular syndrome for which the underlying gene is autoimmune regulator (AIRE). AIRE is a key conditioner of central immunological tolerance by controlling negative selection of T cells in the thymus and inducing a specific subset of regulatory T cells [8]. Mutations in AIRE predispose also to PA similar to the other AIDs manifested in the syndrome. These data suggest that the observed familial clustering may be due to multifactorial polygenic influence.

In the present study, we showed association of PA with 14 other AIDs, and as 10 of these could be confirmed in the bidirectional analyses, the results are likely to represent true associations. Limited data are available on discordant familial associations of PA. Our old family studies on Graves disease, type 1 diabetes, and rheumatoid arthritis detected associations with PA [6, 9, 10]. The study populations were younger than the present one and the familial risks tended to be higher than the current ones. In our recent analysis of thyroid AIDs, we showed that Graves disease and Hashimoto thyroiditis were associated with PA with SIRs of 1.94 and 1.74, respectively; the PA population was younger than the present one [23]. The present results and the older studies show familial risks between PA and such common AIDs which present as comorbidities in the same individuals and which probably indicate underlying genetic susceptibility. However, in addition to rheumatoid arthritis, other rheumatoid AIDs (polymyalgia rheumatica, Sjögren syndrome, and systemic lupus) were associated with PA, as were also other novel associations, including giant-cell arteritis, psoriasis, and sarcoidosis, waiting for confirmation for shared disease mechanisms.

A common limitation of studies on rare diseases is the limited sample size. Although we were able to detect 14 discordant associations, 10 of which were confirmed bidirectionally, many more may have remained undetected due to the rarity of many AIDs. Another possible weakness is that we only included hospitalized patients, but those with mild symptoms may be taken care of in general practice (primary care). A further point is that diagnostic underreporting may be at least a theoretical option; as PA is the end stage of chronic atrophic gastritis, gastritis diagnosis may be rendered first, and PA may remain unreported. However, for the sake of the present analysis, the correctness of the PA diagnosis is most important, and some underreporting should not cause severe bias. A related diagnostic problem is that for the sake of epidemiological design, we only considered the first AID diagnosis. As PA patients experience multiple AID comorbidities, those diagnosed after the first one were not considered in this study. Finally, as the global epidemiology of PA is underdeveloped, we cannot generalize the Swedish results to other population.

## 5. Conclusions

We showed novel evidence for familial risks in concordant PA and for discordant associations of PA with 14 other AIDs. Among 3.6% of the patients, a family member was also diagnosed with PA. The familial risk was two times higher between siblings than between offspring and parents, which may be due to complex genetic background. The discordant associations included some AIDs that have been previously described as comorbidities in PA patients and several yet unreported associations, including rheumatoid AIDs and other AIDs. The result calls for further population-level studies to unravel the mechanisms of familial PA which may lead to further insight into the etiology of PA.

## Figures and Tables

**Table 1:** Number of cases of autoimmune diseases in offspring (*N* = 8.9 million) and in the total population, 1964–2015.

	No. of events in the offspring generation			No. of events in total population	
	No.	%	Mean age	No.	% of males
Total AIDs	612,640		39.8 ± 19.9	896,270	40.2
**Subtype**					
Pernicious anemia	7701	1.3	47.7 ± 18.3	35,906	42.3

**Table 2:** Familial risks of concordant pernicious anemia.

Proband	O	SIR	95% CI		O	SIR	95% CI		O	SIR	95% CI	
	All				Women				Men			
First-degree relative	250	**3.88**	**3.41**	**4.39**	127	**3.58**	**2.98**	**4.26**	123	**4.24**	**3.53**	**5.06**
	Parents only				Sibling only				Both parent and sibling			
Parent/sibling	175	**3.08**	**2.64**	**3.57**	101	**6.43**	**5.24**	**7.82**	2	6.64	0.63	24.43

Bold type: 95% CI does not include 1.00. O = observed number of cases; SIR = standardized incidence ratio; CI = confidence interval.

**Table 3:** Familial risks of discordant autoimmune diseases.

Subtypes of AID in offspring	Family history of AID	Both genders				Men				Women			
		Obs.	SIR	95% CI		Obs.	SIR	95% CI		Obs.	SIR	95% CI	
**Pernicious anemia**	Addison disease	16	**2.45**	**1.40**	**3.99**	7	**2.84**	**1.13**	**5.88**	9	**2.22**	**1.01**	**4.23**
Addison disease	**Pernicious anemia**	29	**1.97**	**1.32**	**2.84**	12	**1.94**	**1.00**	**3.39**	17	**2.00**	**1.16**	**3.22**
**Pernicious anemia**	Celiac disease	68	**1.43**	**1.11**	**1.82**	22	1.33	0.83	2.02	46	**1.49**	**1.09**	**1.98**
Celiac disease	**Pernicious anemia**	227	**1.44**	**1.26**	**1.64**	89	**1.54**	**1.23**	**1.89**	138	**1.38**	**1.16**	**1.63**
**Pernicious anemia**	Crohn disease	136	**1.70**	**1.43**	**2.02**	47	**1.64**	**1.20**	**2.18**	89	**1.74**	**1.40**	**2.14**
Crohn disease	**Pernicious anemia**	265	**1.24**	**1.10**	**1.40**	126	**1.21**	**1.01**	**1.44**	139	**1.28**	**1.07**	**1.51**
**Pernicious anemia**	Diabetes mellitus type I	35	**1.53**	**1.06**	**2.13**	12	1.86	0.96	3.26	23	1.40	0.89	2.10
Diabetes mellitus type I	**Pernicious anemia**	167	**1.74**	**1.48**	**2.02**	86	**1.69**	**1.35**	**2.09**	81	**1.79**	**1.42**	**2.22**
**Pernicious anemia**	Giant-cell arteritis	70	**1.66**	**1.30**	**2.10**	29	**1.65**	**1.10**	**2.37**	41	**1.68**	**1.20**	**2.28**
Giant-cell arteritis	**Pernicious anemia**	89	1.14	0.91	1.40	31	1.11	0.75	1.58	58	1.16	0.88	1.49
**Pernicious anemia**	Graves	201	**1.52**	**1.32**	**1.74**	74	**1.45**	**1.14**	**1.82**	127	**1.56**	**1.30**	**1.86**
Graves	**Pernicious anemia**	424	**1.57**	**1.43**	**1.73**	74	**1.64**	**1.29**	**2.06**	350	**1.56**	**1.40**	**1.73**
**Pernicious anemia**	Hashimoto thyroiditis	130	**1.41**	**1.17**	**1.67**	44	1.28	0.93	1.72	86	**1.48**	**1.18**	**1.83**
Hashimoto thyroiditis	**Pernicious anemia**	266	**1.20**	**1.06**	**1.36**	45	1.23	0.89	1.64	221	**1.20**	**1.05**	**1.37**
**Pernicious anemia**	Polymyalgia rheumatica	82	0.82	0.65	1.02	38	0.86	0.61	1.18	44	0.79	0.57	1.06
Polymyalgia rheumatica	**Pernicious anemia**	146	**1.27**	**1.08**	**1.50**	65	**1.29**	**1.00**	**1.65**	81	**1.26**	**1.00**	**1.57**
**Pernicious anemia**	Psoriasis	413	**1.28**	**1.16**	**1.41**	163	**1.37**	**1.17**	**1.60**	250	**1.23**	**1.08**	**1.39**
Psoriasis	**Pernicious anemia**	957	**1.13**	**1.06**	**1.21**	475	**1.16**	**1.06**	**1.27**	482	**1.11**	**1.01**	**1.21**
**Pernicious anemia**	Rheumatoid arthritis	391	**1.29**	**1.17**	**1.43**	155	**1.25**	**1.06**	**1.46**	236	**1.33**	**1.16**	**1.51**
Rheumatoid arthritis	**Pernicious anemia**	576	**1.21**	**1.12**	**1.32**	157	1.02	0.87	1.19	419	**1.31**	**1.19**	**1.44**
**Pernicious anemia**	Sarcoidosis	63	1.16	0.89	1.48	24	1.13	0.73	1.69	39	1.17	0.83	1.60
Sarcoidosis	**Pernicious anemia**	157	**1.20**	**1.02**	**1.41**	88	1.17	0.94	1.45	69	1.25	0.97	1.58
**Pernicious anemia**	Sjögren syndrome	42	**1.43**	**1.03**	**1.93**	11	0.99	0.49	1.78	31	**1.69**	**1.15**	**2.40**
Sjögren syndrome	**Pernicious anemia**	97	**1.45**	**1.17**	**1.77**	10	1.39	0.66	2.57	87	**1.46**	**1.17**	**1.80**
**Pernicious anemia**	Systemic lupus erythematosus	34	**1.57**	**1.09**	**2.20**	17	**2.01**	**1.17**	**3.23**	17	1.29	0.75	2.06
Systemic lupus erythematosus	**Pernicious anemia**	64	**1.48**	**1.14**	**1.89**	10	1.39	0.66	2.56	54	**1.49**	**1.12**	**1.95**
**Pernicious anemia**	Ulcerative colitis	146	1.07	0.90	1.25	51	1.01	0.75	1.32	95	1.10	0.89	1.35
Ulcerative colitis	**Pernicious anemia**	416	**1.14**	**1.03**	**1.25**	226	**1.15**	**1.00**	**1.31**	190	1.13	0.97	1.30

Bold values indicate that 95% CI does not include 1.00. O = observed number of cases; SIR = standardized incidence ratio; CI = confidence interval.

## Data Availability

The data were obtained from the Swedish National Board of Health and Welfare, to which any request should be addressed.

## Supplementary Materials

Supplementary Materials Supplementary Figure 1: age-specific incidence rate (per 1, 00, 000 person years) of pernicious anemia in Sweden, 1964–2015. Supplementary Figure 2: age-specific prevalence (per 1000 person years) of pernicious anemia in Sweden in 2010–2015. Supplementary Table 1: hospitalizations for autoimmune disorders in Sweden, 1964–2015. Supplementary Table 2: familial risks for pernicious anemia with discordant autoimmune diseases which did not reach statistical significance.
