# Supplementary material for: Familial Risks between Pernicious Anemia and Other Autoimmune Diseases in the Population of Sweden
Source: Autoimmune Dis. 2021 Jan 12;2021:8815297. doi: 10.1155/2021/8815297 (PMC7815416; doi:10.1155/2021/8815297)

FAMILIAL RISKS BETWEEN PERNICIOUS ANEMIA AND OTHER AUTOIMMUNE DISEASES IN THE POPULATION OF SWEDEN

**SUPPLEMENTARY MATERIAL**

Xinjun Li^1^, Hauke Thomsen^1,2,9^, Kristina Sundquist^1,3,4^, Jan Sundquist^1,3,4^, Asta Försti^1,2,5,6^, Kari Hemminki^1,2,7,8^

^1^Center for Primary Health Care Research, Lund University, Malmö, Sweden;

^2^Division of Molecular Genetic Epidemiology, German Cancer Research Centre (DKFZ), 69120 Heidelberg, Germany;

^3^Department of Family Medicine and Community Health, Department of Population Health Science and Policy, Icahn School of Medicine at Mount Sinai, New York, USA

^4^Center for Community-based Healthcare Research and Education (CoHRE), Department of Functional Pathology, School of Medicine, Shimane University, Japan

5Hopp Children's Cancer Center (KiTZ), Heidelberg, Germany

^6^Division of Pediatric Neurooncology, German Cancer Research Center (DKFZ), German Cancer Consortium (DKTK), Heidelberg, Germany

^7^Division of Cancer Epidemiology, German Cancer Research Centre (DKFZ), 69120 Heidelberg, Germany;

^8^Faculty of Medicine and Biomedical Center in Pilsen, Charles University in Prague, 30605 Pilsen, Czech Republic

^9^Genewerk GmbH, Heidelberg, Germany

Corresponding Author: Hauke Thomsen,

GeneWerk GmbH, Im Neuenheimer Feld 582, D-69120 Heidelberg, Germany

Phone: +49 (0) 6221 42790-24

Fax: +49 (0) 6221 42790-12

hauke.thomsen@genewerk.de

**Supplementary Table 1: Hospitalizations for autoimmune disorders in Sweden,**

**1964-2015**

|  |  | Hospitalizations | |
| --- | --- | --- | --- |
| **Autoimmune condition** |  | No. | % |
| Addison’s disease |  | 3213 | 0.4 |
| Amyotrophic lateral sclerosis |  | 10,817 | 1.2 |
| Angiitis hypersensitiva |  | 359 | 0.0 |
| Ankylosing spondylitis |  | 17,206 | 1.9 |
| Autoimmune hemolytic anemia |  | 1461 | 0.2 |
| Behçet’s disease |  | 935 | 0.1 |
| Celiac disease |  | 45,886 | 5.1 |
| Chorea minor |  | 160 | 0.0 |
| Chronic rheumatic heart disease |  | 24,656 | 2.8 |
| Crohn’s disease |  | 41,627 | 4.6 |
| Dermatitis Herpetiformis |  | 2466 | 0.3 |
| Diabetes mellitus type I |  | 29,854 | 3.3 |
| Discoid lupus erythematosus |  | 3891 | 0.4 |
| Giant-cell arteritis |  | 15,946 | 1.8 |
| Glomerular nephritis acute |  | 17,958 | 2.0 |
| Glomerular nephritis chronic |  | 8593 | 1.0 |
| Graves’ disease |  | 60,673 | 6.8 |
| Guillain-Barré syndrome |  | 4558 | 0.5 |
| Hashimoto’s thyroiditis |  | 52,271 | 5.8 |
| Immune thrombocytopenic purpura |  | 12911 | 1.4 |
| Localized scleroderma |  | 3282 | 0.4 |
| Lupoid hepatitis |  | 6856 | 0.8 |
| Multiple sclerosis |  | 29,263 | 3.3 |
| Myasthenia gravis |  | 4579 | 0.5 |
| Pemphigoid |  | 8071 | 0.9 |
| Pemphigus |  | 1639 | 0.2 |
| Pernicious anemia |  | 35,906 | 4.0 |
| Polyarteritis nodosa |  | 1722 | 0.2 |
| Polymyalgia rheumatica |  | 40,697 | 4.5 |
| Polymyositis/dermatomyositis |  | 2974 | 0.3 |
| Primary biliary cirrhosis |  | 4761 | 0.5 |
| Psoriasis |  | 150,979 | 16.8 |
| Reiter’s disease |  | 1612 | 0.2 |
| Rheumatic fever |  | 4197 | 0.5 |
| Rheumatoid arthritis |  | 121,723 | 13.6 |
| Sarcoidosis |  | 23,847 | 2.7 |
| Sjögren’s syndrome |  | 11,091 | 1.2 |
| Systemic lupus erythematosus |  | 10,198 | 1.1 |
| Systemic sclerosis |  | 3753 | 0.4 |
| Takayasus disease |  | 423 | 0.0 |
| Thrombotic thrombocytopenic purpura |  | 499 | 0.1 |
| Ulcerative colitis |  | 69,428 | 7.7 |
| Wegener’s granulomatosis |  | 3329 | 0.4 |
| All above |  | 896,270 | 100.0 |

| **Supplementary Table 2: Familial risks for pernicious anemia with disconcordant autoimmune diseases which did not reach statistical signifcance** | | | | | | | | | | | | | | | |
| --- | --- | --- | --- | --- | --- | --- | --- | --- | --- | --- | --- | --- | --- | --- | --- |
| Subtypes of AID in offspring | Family history of AID | Both genders | | | |  | Men | | | |  | Women | | | |
|  |  | Obs. | SIR | 95% CI | |  | Obs. | SIR | 95% CI | |  | Obs. | SIR | 95% CI | |
| **Pernicious anemia** | Amyotrophic lateral sclerosis | 24 | 0.88 | 0.57 | 1.32 |  | 7 | 0.61 | 0.24 | 1.26 |  | 17 | 1.08 | 0.63 | 1.74 |
| Amyotrophic lateral sclerosis | **Pernicious anemia** | 39 | 0.96 | 0.68 | 1.31 |  | 26 | 1.03 | 0.67 | 1.52 |  | 13 | 0.84 | 0.44 | 1.43 |
|  |  |  |  |  |  |  |  |  |  |  |  |  |  |  |  |
| **Pernicious anemia** | Angiitis hypersensitive | 3 | 4.17 | 0.79 | 12.35 |  | 1 | 3.44 | 0.00 | 19.72 |  | 2 | 4.67 | 0.44 | 17.18 |
| Angiitis hypersensitive | **Pernicious anemia** | 2 | 1.35 | 0.13 | 4.97 |  | 0 |  |  |  |  | 2 | 2.37 | 0.22 | 8.71 |
|  |  |  |  |  |  |  |  |  |  |  |  |  |  |  |  |
| **Pernicious anemia** | Ankylosing spondylitis | 33 | 0.98 | 0.67 | 1.38 |  | 9 | 0.75 | 0.34 | 1.44 |  | 24 | 1.11 | 0.71 | 1.65 |
| Ankylosing spondylitis | **Pernicious anemia** | 80 | 0.89 | 0.70 | 1.10 |  | 51 | 0.82 | 0.61 | 1.08 |  | 29 | 1.02 | 0.68 | 1.46 |
|  |  |  |  |  |  |  |  |  |  |  |  |  |  |  |  |
| **Pernicious anemia** | Autoimmune hemolytic anemia | 2 | 0.70 | 0.07 | 2.58 |  | 1 | 0.82 | 0.00 | 4.71 |  | 1 | 0.61 | 0.00 | 3.52 |
| Autoimmune hemolytic anemia | **Pernicious anemia** | 6 | 1.90 | 0.68 | 4.15 |  | 5 | 3.31 | 1.04 | 7.78 |  | 1 | 0.60 | 0.00 | 3.47 |
|  |  |  |  |  |  |  |  |  |  |  |  |  |  |  |  |
| **Pernicious anemia** | Behcet disease | 0 |  |  |  |  | 0 |  |  |  |  | 0 |  |  |  |
| Behcet disease | **Pernicious anemia** | 10 | 2.60 | 1.24 | 4.80 |  | 4 | 2.47 | 0.64 | 6.39 |  | 6 | 2.70 | 0.97 | 5.90 |
|  |  |  |  |  |  |  |  |  |  |  |  |  |  |  |  |
| **Pernicious anemia** | Chorea minor | 0 |  |  |  |  | 0 |  |  |  |  | 0 |  |  |  |
| Chorea minor | **Pernicious anemia** | 0 |  |  |  |  | 0 |  |  |  |  | 0 |  |  |  |
|  |  |  |  |  |  |  |  |  |  |  |  |  |  |  |  |
| **Pernicious anemia** | Chronic rheumatic heart disease | 59 | 1.13 | 0.86 | 1.46 |  | 24 | 1.04 | 0.67 | 1.56 |  | 35 | 1.20 | 0.84 | 1.67 |
| Chronic rheumatic heart disease | **Pernicious anemia** | 31 | 0.85 | 0.58 | 1.21 |  | 19 | 0.90 | 0.54 | 1.41 |  | 12 | 0.78 | 0.40 | 1.36 |
|  |  |  |  |  |  |  |  |  |  |  |  |  |  |  |  |
| **Pernicious anemia** | Dermatitis herpetiformis | 9 | 1.63 | 0.74 | 3.10 |  | 3 | 1.29 | 0.24 | 3.83 |  | 6 | 1.87 | 0.67 | 4.09 |
| Dermatitis herpetiformis | **Pernicious anemia** | 12 | 1.02 | 0.53 | 1.79 |  | 7 | 1.06 | 0.42 | 2.20 |  | 5 | 0.98 | 0.31 | 2.30 |
|  |  |  |  |  |  |  |  |  |  |  |  |  |  |  |  |
| **Pernicious anemia** | Discoid lupus erythematosus | 10 | 1.06 | 0.50 | 1.95 |  | 2 | 0.58 | 0.05 | 2.13 |  | 8 | 1.33 | 0.57 | 2.64 |
| Discoid lupus erythematosus | **Pernicious anemia** | 27 | 1.27 | 0.83 | 1.85 |  | 10 | 1.78 | 0.85 | 3.28 |  | 17 | 1.08 | 0.63 | 1.74 |
|  |  |  |  |  |  |  |  |  |  |  |  |  |  |  |  |
| **Pernicious anemia** | Glomerular nephritis chronic | 31 | 0.88 | 0.60 | 1.26 |  | 9 | 0.64 | 0.29 | 1.22 |  | 22 | 1.05 | 0.66 | 1.59 |
| Glomerular nephritis chronic | **Pernicious anemia** | 72 | 0.97 | 0.76 | 1.22 |  | 51 | 1.04 | 0.77 | 1.36 |  | 21 | 0.83 | 0.51 | 1.27 |
|  |  |  |  |  |  |  |  |  |  |  |  |  |  |  |  |
| **Pernicious anemia** | Glomerular nephritis acute | 13 | 0.84 | 0.44 | 1.44 |  | 5 | 0.87 | 0.27 | 2.04 |  | 8 | 0.82 | 0.35 | 1.62 |
| Glomerular nephritis acute | **Pernicious anemia** | 37 | 0.90 | 0.63 | 1.24 |  | 25 | 0.96 | 0.62 | 1.41 |  | 12 | 0.80 | 0.41 | 1.41 |
|  |  |  |  |  |  |  |  |  |  |  |  |  |  |  |  |
| **Pernicious anemia** | Guillain-Barre Syndrome | 13 | 1.31 | 0.69 | 2.24 |  | 3 | 0.79 | 0.15 | 2.34 |  | 10 | 1.62 | 0.77 | 3.00 |
| Guillain-Barre Syndrome | **Pernicious anemia** | 29 | 1.20 | 0.80 | 1.72 |  | 18 | 1.23 | 0.73 | 1.94 |  | 11 | 1.15 | 0.57 | 2.07 |
|  |  |  |  |  |  |  |  |  |  |  |  |  |  |  |  |
| **Pernicious anemia** | Immune thrombocytopenic purpura | 22 | 1.02 | 0.64 | 1.55 |  | 10 | 1.23 | 0.59 | 2.27 |  | 12 | 0.90 | 0.46 | 1.57 |
| Immune thrombocytopenic purpura | **Pernicious anemia** | 42 | 0.86 | 0.62 | 1.16 |  | 19 | 0.78 | 0.47 | 1.22 |  | 23 | 0.93 | 0.59 | 1.40 |
|  |  |  |  |  |  |  |  |  |  |  |  |  |  |  |  |
| **Pernicious anemia** | Localized scleroderma | 7 | 1.06 | 0.42 | 2.19 |  | 4 | 1.61 | 0.42 | 4.16 |  | 3 | 0.73 | 0.14 | 2.15 |
| Localized scleroderma | **Pernicious anemia** | 14 | 0.80 | 0.43 | 1.34 |  | 5 | 0.95 | 0.30 | 2.23 |  | 9 | 0.73 | 0.33 | 1.39 |
|  |  |  |  |  |  |  |  |  |  |  |  |  |  |  |  |
| **Pernicious anemia** | Lupoid hepatitis | 15 | 0.97 | 0.54 | 1.60 |  | 8 | 1.33 | 0.57 | 2.63 |  | 7 | 0.74 | 0.29 | 1.53 |
| Lupoid hepatitis | **Pernicious anemia** | 33 | 1.00 | 0.69 | 1.41 |  | 13 | 0.95 | 0.50 | 1.63 |  | 20 | 1.04 | 0.63 | 1.60 |
|  |  |  |  |  |  |  |  |  |  |  |  |  |  |  |  |
| **Pernicious anemia** | Multiple sclerosis | 58 | 0.96 | 0.73 | 1.25 |  | 23 | 1.03 | 0.65 | 1.55 |  | 35 | 0.93 | 0.65 | 1.29 |
| Multiple sclerosis | **Pernicious anemia** | 188 | 1.14 | 0.98 | 1.31 |  | 59 | 1.10 | 0.84 | 1.42 |  | 129 | 1.16 | 0.97 | 1.37 |
|  |  |  |  |  |  |  |  |  |  |  |  |  |  |  |  |
| **Pernicious anemia** | Myasthenia gravis | 17 | 1.61 | 0.94 | 2.58 |  | 7 | 1.65 | 0.65 | 3.41 |  | 10 | 1.59 | 0.75 | 2.93 |
| Myasthenia gravis | **Pernicious anemia** | 19 | 0.95 | 0.57 | 1.49 |  | 10 | 1.05 | 0.50 | 1.94 |  | 9 | 0.86 | 0.39 | 1.64 |
|  |  |  |  |  |  |  |  |  |  |  |  |  |  |  |  |
| **Pernicious anemia** | Pemphigoid | 15 | 0.94 | 0.53 | 1.56 |  | 4 | 0.59 | 0.15 | 1.52 |  | 11 | 1.21 | 0.60 | 2.17 |
| Pemphigoid | **Pernicious anemia** | 17 | 1.00 | 0.58 | 1.60 |  | 8 | 0.91 | 0.39 | 1.79 |  | 9 | 1.10 | 0.50 | 2.10 |
|  |  |  |  |  |  |  |  |  |  |  |  |  |  |  |  |
| **Pernicious anemia** | Pemphigus | 3 | 1.05 | 0.20 | 3.10 |  | 1 | 0.89 | 0.00 | 5.12 |  | 2 | 1.15 | 0.11 | 4.22 |
| Pemphigus | **Pernicious anemia** | 2 | 0.37 | 0.03 | 1.37 |  | 0 |  |  |  |  | 2 | 0.74 | 0.07 | 2.71 |
|  |  |  |  |  |  |  |  |  |  |  |  |  |  |  |  |
| **Pernicious anemia** | Polyarteritis nodosa | 8 | 2.15 | 0.92 | 4.25 |  | 4 | 2.53 | 0.66 | 6.55 |  | 4 | 1.86 | 0.48 | 4.81 |
| Polyarteritis nodosa | **Pernicious anemia** | 12 | 2.41 | 1.24 | 4.22 |  | 4 | 1.65 | 0.43 | 4.28 |  | 8 | 3.13 | 1.34 | 6.19 |
|  |  |  |  |  |  |  |  |  |  |  |  |  |  |  |  |
| **Pernicious anemia** | Polymyositis/dermatomyositis | 12 | 1.62 | 0.83 | 2.84 |  | 6 | 2.09 | 0.75 | 4.57 |  | 6 | 1.33 | 0.48 | 2.91 |
| Polymyositis/dermatomyositis | **Pernicious anemia** | 15 | 1.03 | 0.57 | 1.70 |  | 6 | 0.95 | 0.34 | 2.08 |  | 9 | 1.09 | 0.50 | 2.08 |
|  |  |  |  |  |  |  |  |  |  |  |  |  |  |  |  |
| **Pernicious anemia** | Primary biliary cirrhosis | 10 | 0.86 | 0.41 | 1.59 |  | 3 | 0.64 | 0.12 | 1.89 |  | 7 | 1.02 | 0.40 | 2.11 |
| Primary biliary cirrhosis | **Pernicious anemia** | 30 | 1.45 | 0.97 | 2.07 |  | 5 | 1.40 | 0.44 | 3.28 |  | 25 | 1.46 | 0.94 | 2.15 |
|  |  |  |  |  |  |  |  |  |  |  |  |  |  |  |  |
| **Pernicious anemia** | Reiter disease | 5 | 1.28 | 0.41 | 3.02 |  | 2 | 1.42 | 0.13 | 5.21 |  | 3 | 1.21 | 0.23 | 3.58 |
| Reiter disease | **Pernicious anemia** | 5 | 0.47 | 0.15 | 1.12 |  | 4 | 0.43 | 0.11 | 1.12 |  | 1 | 0.77 | 0.00 | 4.42 |
|  |  |  |  |  |  |  |  |  |  |  |  |  |  |  |  |
| **Pernicious anemia** | Rheumatic fever | 10 | 1.02 | 0.49 | 1.89 |  | 6 | 1.56 | 0.56 | 3.41 |  | 4 | 0.68 | 0.18 | 1.75 |
| Rheumatic fever | **Pernicious anemia** | 18 | 0.93 | 0.55 | 1.47 |  | 12 | 0.80 | 0.41 | 1.40 |  | 6 | 1.36 | 0.49 | 2.99 |
|  |  |  |  |  |  |  |  |  |  |  |  |  |  |  |  |
| **Pernicious anemia** | Systemic sclerosis | 9 | 0.98 | 0.45 | 1.87 |  | 3 | 0.84 | 0.16 | 2.49 |  | 6 | 1.07 | 0.39 | 2.35 |
| Systemic sclerosis | **Pernicious anemia** | 20 | 1.15 | 0.70 | 1.78 |  | 5 | 1.25 | 0.40 | 2.95 |  | 15 | 1.12 | 0.63 | 1.86 |
|  |  |  |  |  |  |  |  |  |  |  |  |  |  |  |  |
| **Pernicious anemia** | Takayasu disease | 0 |  |  |  |  | 0 |  |  |  |  | 0 |  |  |  |
| Takayasu disease | **Pernicious anemia** | 2 | 0.92 | 0.09 | 3.38 |  | 1 | 1.59 | 0.00 | 9.09 |  | 1 | 0.65 | 0.00 | 3.72 |
|  |  |  |  |  |  |  |  |  |  |  |  |  |  |  |  |
| **Pernicious anemia** | Thrombotic thrombocytop | 1 | 0.99 | 0.00 | 5.66 |  | 1 | 2.78 | 0.00 | 15.96 |  | 0 |  |  |  |
| Thrombotic thrombocytop | **Pernicious anemia** | 1 | 0.48 | 0.00 | 2.75 |  | 1 | 0.98 | 0.00 | 5.61 |  | 0 |  |  |  |
|  |  |  |  |  |  |  |  |  |  |  |  |  |  |  |  |
| **Pernicious anemia** | Wegener granulomatosis | 9 | 1.13 | 0.51 | 2.16 |  | 3 | 0.99 | 0.19 | 2.93 |  | 6 | 1.22 | 0.44 | 2.67 |
| Wegener granulomatosis | **Pernicious anemia** | 19 | 1.08 | 0.65 | 1.69 |  | 9 | 0.88 | 0.40 | 1.67 |  | 10 | 1.37 | 0.65 | 2.53 |

AID=Autoimmune disorders; SIR=Standardized incidence ratio; CI=Confidence interval.

**Supplementary Figure legends**

**Supplementary Figure 1. Age-specific incidence rate (per 100 000 person years) of pernicious anemia in Sweden, 1964-2015**

Total incidence rate (European standardized population) =8.3 per 100 000 person years, males=7.5 per 100 000 person years, and females=9.1 per 100 000 person years.

**Supplementary Figure 2. Age-specific prevalence (per 1000 person years) of pernicious anemia in Sweden in 2010-2015**

Total prevalence (European standardized population) =0.9 per 1000, males=0.8 per 1000, and females=1.1 per 1000.


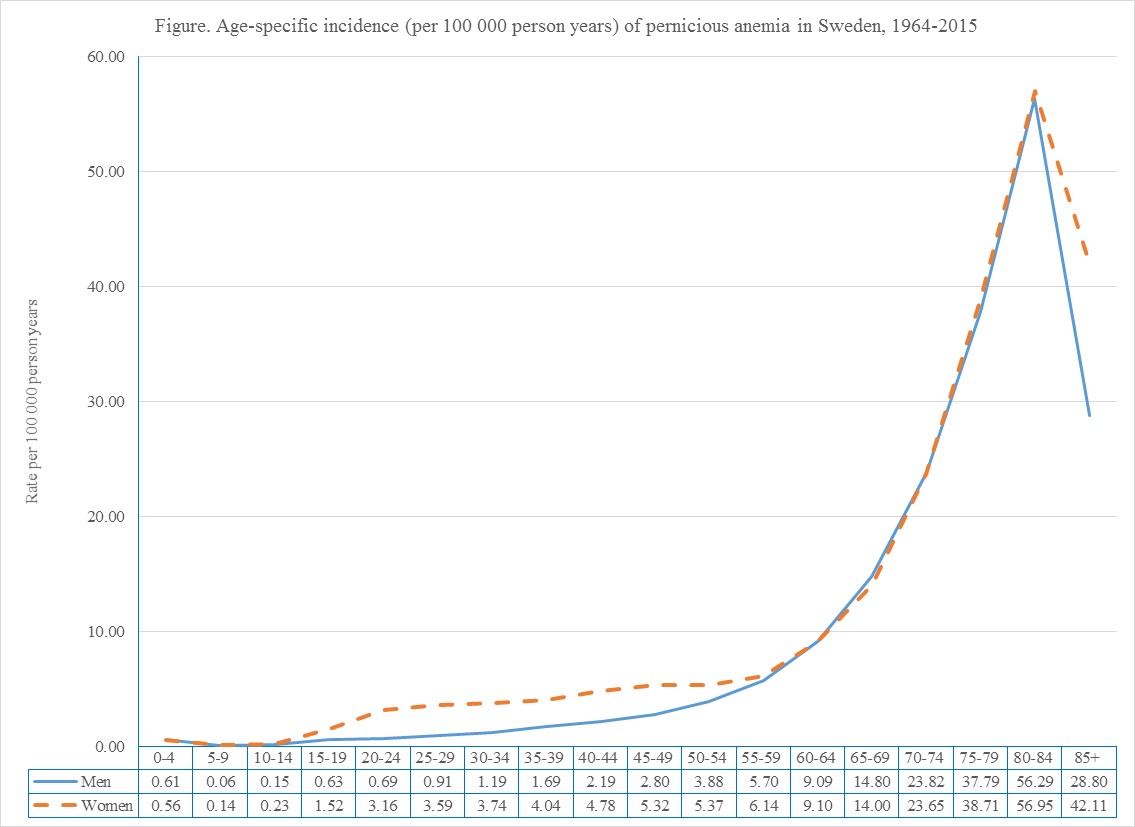


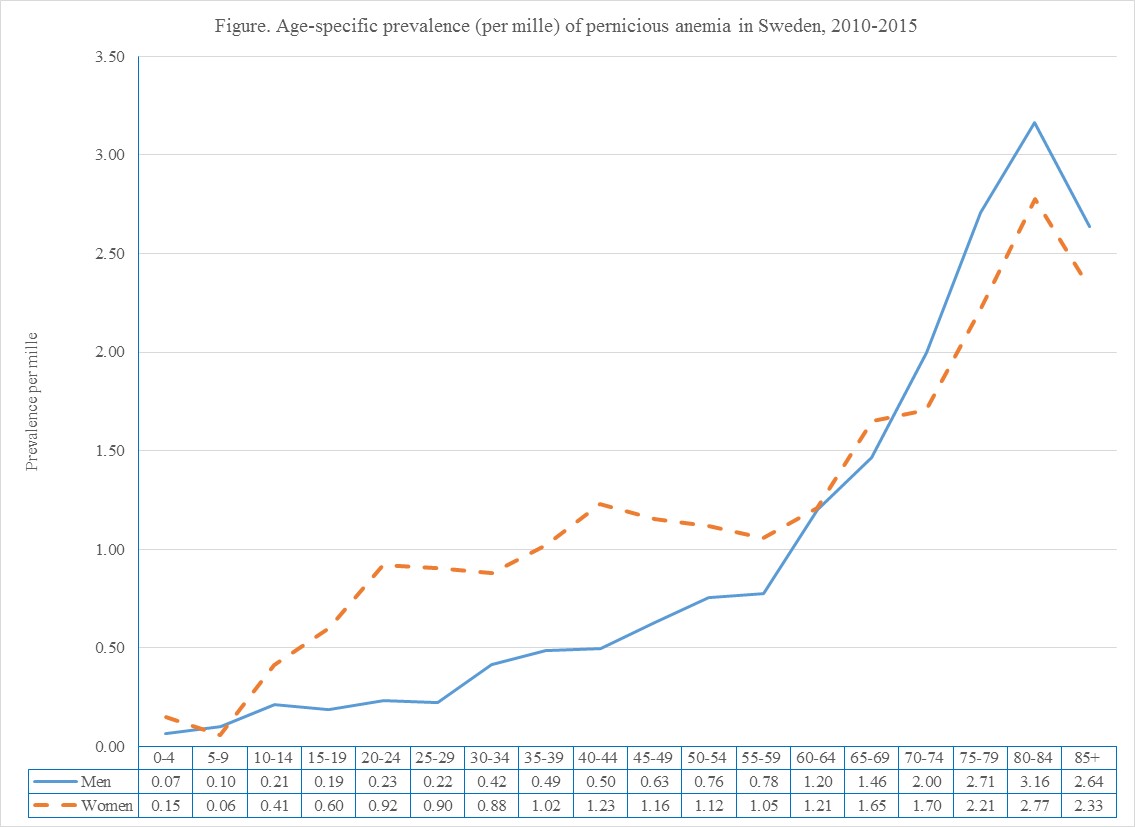

Supplement: Supplementary Materials — Supplementary Figure 1: age-specific incidence rate (per 1, 00, 000 person years) of pernicious anemia in Sweden, 1964–2015. Supplementary Figure 2: age-specific prevalence (per 1000 person years) of pernicious anemia in Sweden in 2010–2015. Supplementary Table 1: hospitalizations for autoimmune disorders in Sweden, 1964–2015. Supplementary Table 2: familial risks for pernicious anemia with discordant autoimmune diseases which did not reach statistical significance. [file 8815297.f1.docx]
